# Highly sensitive multipoint real-time kinetic detection of Surface Plasmon bioanalytes with custom CMOS cameras

**DOI:** 10.1016/j.bios.2014.02.042

**Published:** 2014-08-15

**Authors:** Jing Wang, Richard J. Smith, Roger A. Light, Joanna L. Richens, Jing Zhang, Paul O’Shea, Chung See, Michael G. Somekh

**Affiliations:** Institute of Biophysics, Imaging and Optical Science, Department of Electrical and Electronic Engineering, University of Nottingham, Nottinghamshire NG7 2RD, United Kingdom

**Keywords:** Surface plasmon resonance, Phase sensitive, Multipoint detection, High sensitivity

## Abstract

Phase sensitive Surface Plasmon Resonance (SPR) techniques are a popular means of characterizing biomolecular interactions. However, limitations due to the narrow dynamic range and difficulty in adapting the method for multi-point sensing have restricted its range of applications. This paper presents a compact phase sensitive SPR technology using a custom CMOS camera. The system is exceptionally versatile enabling one to trade dynamic range for sensitivity without altering the optical system. We present results showing sensitivity over the array of better than 10^−6^ Refractive Index Units (RIU) over a refractive index range of 2×10^−2^ RIU, with peak sensitivity of 3×10^−7^ RIU at the center of this range. We also explain how simply altering the settings of polarization components can give sensitivity on the order of 10^−8^ RIU albeit at the cost of lower dynamic range. The consistent response of the custom CMOS camera in the system also allowed us to demonstrate precise quantitative detection of two Fibrinogen antibody–protein binding sites. Moreover, we use the system to determine reaction kinetics and argue how the multipoint detection gives useful insight into the molecular binding mechanisms.

## Highlights

• We introduced a SPR sensor with an optimal sensitivity of 3×10^−7^ RIU.
• Sensitivity better than 10^−6^ RIU was achieved for refractive index 1.33 ~1.35 RIU.
• Relation between responsivity and dynamic range was illustrated.
• Potential performance (sensitivity of 4.7×10^−8^ RIU) was estimated.
• New AC detector enabling multi-point sensing was introduced.

## 1 Introduction

Surface Plasmon Resonance (SPR) using antibody capture is a preferred technique for the detection of bioanalytes, in, instance, disease diagnosis, due to its high sensitivity and label free nature (Schunck, 1997; Schasfoort and Tudos, 2008). There are several challenges that need to be addressed in the preparation of biological samples. These involve the separation of analytes and the desire to detect the biomarkers in plasma, which, in turn, involves the development of protocols to overcome non-specific binding. There are a separate set of instrumentation engineering challenges that we address in the present paper. We summarize three key desirable features of the instrumentation:

(A) *High sensitivity detection capability in the range* 10^−7^ RIU: Sensitivity around 10^−7^ RIU approaches the sensitivity of commercial instruments using single point detection (Abdiche et al., 2009) and this allows one to measure binding kinetics of analytes with relatively low concentration and/or molecular weight (Karlsson, 2004; Copper, 2002).
(B) *Multipoint detection*: Many disease types are not well defined by a single marker and require the coexistence of a panel of markers to ensure reliability of measurement (Vafadar-Isfahani et al., 2012).
(C) *Large measurement range covering between* 10^−2^ *and* 4×10^−2^ RIU: In a single binding measurement the change in the index of the measured region during an experiment only varies by a few hundred µRIU, however, variations in the background index give a much larger sample to sample variation; so large measurement range means that readjustment of the system is not necessary between samples.

There have been many attempts to achieve high sensitivity for SPR sensors involving measurement of both the amplitude (Piliarik and Homola, 2009; Shumaker-Parry and Campbell, 2004; Fu et al., 2004; Nenninger et al., 2002) and phase (Kabashin and Nikitin, 1997; Grigorenko et al., 1999; Wu et al., 2004; Notcovich et al., 2000) of the Surface Plasmon (SP) signal. Although, still the subject of debate, it is generally accepted that phase measurements give better sensitivity compared to amplitude only approaches (Kabashin et al., 2009). The problem with phase measurements is that the sharp phase change that leads to the high sensitivity means that the dynamic range is generally poor unless measures are taken which increase the complexity of the system; one example is the use of white light spectral interferometry (Ng et al., 2011). This complexity means that it is challenging to combine high sensitivity and high dynamic range with multipoint imaging. Another technique that uses the phase of the signal is differential surface plasmon ellipsometry (Hooper et al., 2006; Hooper and Sambles, 2006) in which an interferometer is formed between the input p- and s-polarizations. Changes in the SP reflection conditions affect the ellipticity of the reflected beam and by dithering the polarization of the input beam the output signal oscillates at the dither frequency and harmonics thereof; this allows either the fundamental or the first harmonic to be detected in a lock-in amplifier. This technique gives excellent sensitivity and, as we will show later in this paper, gives dynamic range comparable to white light interferometry (Ng et al., 2011). High throughput multi-point detection has been achieved by a number of different approaches (Bardin et al., 2009; Hemmi et al., 2013). Early examples using amplitude configurations with CCD cameras had sensitivity of around 10^−5^ RIU (Fu et al., 2004). Wavelength multiplexing has allowed increased sensitivity but at increased complexity (Johansen et al., 2000; Wong et al., 2006). Development of common path phase sensitive configurations has increased the sensitivity considerably (Homola, 2008; Wang et al., 2012) but generally with a reduced dynamic range. These techniques have increased the number of simultaneous measurements from single or a few points to ‘several hundred’ (Homola, 2008). The method we adopt in this paper is well suited for array imaging on account of the fact that once the system is aligned the sample is illuminated at a single angle of incidence. The problem from the point of view of multipoint imaging is that the oscillating output requires a sensitive detector array capable of detecting modulated light. Here we use a custom CMOS detector to perform multipoint measurements with sensitivity comparable to values obtained with single point measurements.

Prevously, we presented proof of concept experiments (Hooper et al., 2006) to show that differential SP ellipsometry combined with a custom CMOS detector could measure changes in refractive index at many points simultaneously. Changes in refractive index of developed photoresist in air were measured over a two dimensional array. In the present paper we take a similar approach but make a step change in the practical usefulness of the method.

A. *The previous detector array used continuous time electronics which limited the achievable sensitivity*; *here the use of discrete sampling achieves shot noise limited detection so that the sensitivity at each pixel is similar to that achievable with a single point measurement*.
B. *We now show the effectiveness of multipoint imaging in a realistic aqueous environment and quantify the refractive index sensitivity*.
C. *We show the effectiveness of the combined optical and camera system to monitor the spatial distribution of protein binding to antibodies in real time*.

## 2 Materials and methods

### 2.1 Instrument theory and background

**Fig. 1:**
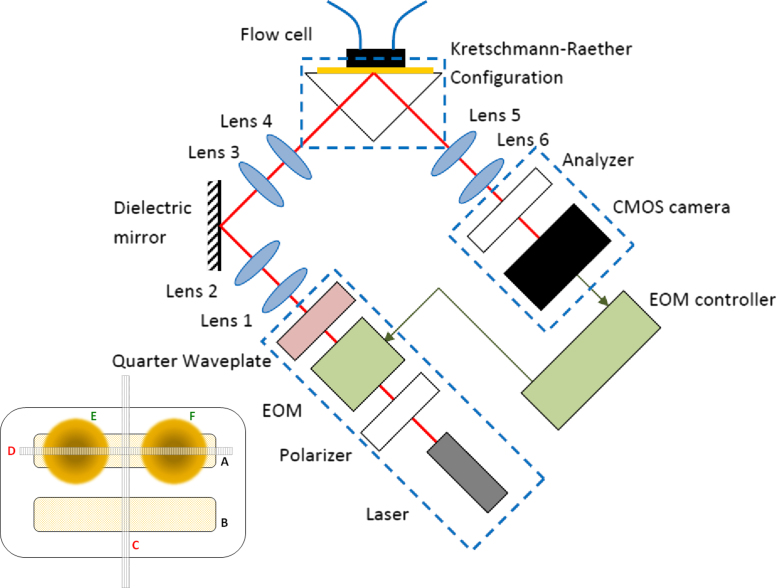
Schematic of SPR imaging system, the boxes show the key functional units. The focal lengths of lenses from 1 to 6 are 20, 100, 30, 30, 40, and 30 mm, respectively. (inset) Schematic of alignment of the flow cell and linear camera. A and B: two channels where liquids flow through; C: alignment of the camera for experiments described in Figs. 3 and 5; D: alignment of the camera for experiments in Fig. 6. E and F indicated the position of two antibody droplets in channel A for experiments in Fig. 6.

Fig. 1a shows the optical system used in the present study. To understand the operation of the system it is necessary to appreciate the functions of the composite units enclosed in the boxes. In Section 2.2 we discuss the specific choice of components within those boxes. The system consists of a light source (assumed monochromatic), a polarization modulator unit to dither the input polarization, projection optics to illuminate the gold coated prism and further projection optics to the optical detector capable of detecting the modulated component of the returning light. The modulated light detector and the polarization modulator unit are frequency locked to each other to give coherent detection.

After the polarization modulator unit the beam was linearly polarized with the polarization angle varying sinusoidally at frequency, *f*. The optical arrangement allows the mean value of the incident polarization angle, *ϕ*, in Eqs. (1)–(3), to be varied. This was incident on the gold surface at or close to the surface plasmon resonance angle and was then directed to the analyzer and modulated light camera. The signals detected by the camera can be derived by describing the system in terms of Jones matrices (Stewart et al., 2008). The resulting equations for the DC, fundamental (1*f*) and second harmonics (2*f*) are given in Eqs. (1)–(3).

(1) $$DC=R_{p}\cos^{2}\psi +R_{s}\sin^{2}\psi +J_{0}(\Delta )\{\;\cos\;2\phi (R_{p}\cos^{2}\Psi -R_{s}\sin^{2}\Psi )+M\;\sin\;2\psi \;\sin\;2\phi \}$$

(2) $$1f=2J_{1}(\Delta )\{\;\sin\;2\phi (R_{s}\sin^{2}\Psi -R_{p}\cos^{2}\Psi )+M\;\sin\;2\psi \;\cos\;2\phi \}$$

(3) $$2f=2J_{2}(\Delta )\{\;\cos\;2\phi (R_{p}\cos^{2}\Psi -R_{s}\sin^{2}\Psi )+M\;\sin\;2\psi \;\sin\;2\phi \}$$

Reflectivity of p-polarization at prism/gold interface:

$$r_{p}=r_{pr}+i\cdot r_{pi}=\frac{n_{Au}\;\cos\;\theta_{i}-n_{pr}\;\cos\;\theta_{t}}{n_{pr}\;\cos\;\theta_{t}+n_{Au}\;\cos\;\theta_{i}};$$

Reflectivity of s-polarization at prism/gold interface:

$$r_{s}=r_{sr}+i\cdot r_{si}=\frac{n_{pr}\;\cos\;\theta_{i}-n_{Au}\;\cos\;\theta_{t}}{n_{pr}\;\cos\;\theta_{i}+n_{Au}\;\cos\;\theta_{t}};$$

where

$n_{Au}$ and $n_{pr}$: refractive index of gold and prism, respectively;
$\theta_{i}$ and $\theta_{t}$: incident angle and refracted angle at prism/gold interface;

$$M=r_{pr}r_{sr}+r_{pi}r_{si};$$

$R_{p}={\left|r_{p}\right|}^{2}$: Reflective intensity of p-polarization;
$R_{s}={\left|r_{s}\right|}^{2}$: Reflective intensity of s-polarization;
Δ: Amplitude of sinusoidal phase modulation of EOM which is $\Delta \;\sin(\omega_{EOM}t)$;
$\omega_{EOM}$: Angular modulation frequency of EOM which is locked to the CMOS camera;
$J_{1}(\Delta )$ and $J_{2}(\Delta )$: first and second order of Bessel function;
ϕ: Polarization angle of input polarizer and the quarter wave plate.
Ψ: Polarization angle of output analyzer.

These equations predict the expected response of the instrument to changes in sample refractive indices. Similar equations were presented by Stewart et al. (2008), however, there appears to be a typographical error in their expression for the 2*f* term.

The responsivity of modulation depth (1*f* peak to peak amplitude divided by DC) to changes in the refractive index at the interface of the gold layer was simulated for different configurations. The responsivity was defined as the change in modulation index per refractive index unit. The sensitivity of the whole system is discussed in Section 3.2 which takes into account both the responsivity and system noise.

**Fig. 2:**
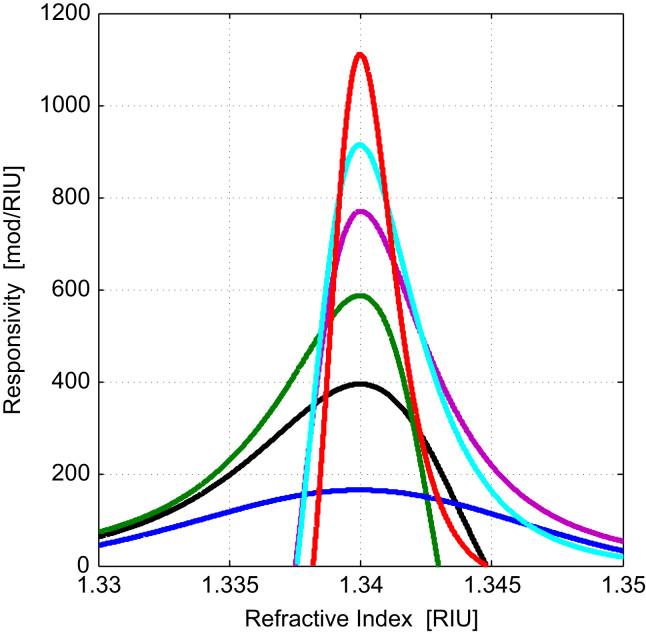
Responsivity in units of modulation depth per RIU for different system parameter configurations, where the maximum change in modulation depth with refractive index for was set to 1.34 RIU. The wavelength was 633 nm and the gold thickness was 50 nm. By changing the polarizer angle ϕ and analyzer angle Ψ, the responsivity varies in maximum value and dynamic range with refractive index. Red:ϕ:30,Ψ 172, Cyan: ϕ:40,Ψ 168, purple: ϕ:50, Ψ 168, Green: ϕ:60, Ψ 167, Black: ϕ:70, Ψ 162, Blue: ϕ:80, Ψ 144. (For interpretation of the references to color in this figure legend, the reader is referred to the web version of this article.)

Fig. 2 shows the effect of varying the polarizer and analyzer angle for a fixed incidence angle and wavelength of 633 nm (for clarity the curves have been centered on an index of 1.34 by making small changes to incident angle for each curve typically less than 0.3°). We can see that the responsivity is highly dependent on the settings and a responsivity of 940 RIU^−1^ is shown (cyan curve), theoretically even greater responsivities (red curve) can be achieved but only when the amount of background light was very low. For this reason for all the curves (apart from the red) we restrict the responsivity calculations to situations where the reflected light intensity was at least 5% of the incident intensity. When the responsivity is high the range over which a sensitive measurement is achieved (dynamic range) is small. For this reason a compromise between dynamic range and responsivity was used throughout this paper as discussed below. The ability to trade sensitivity for dynamic range with the same the optical system is an especially powerful feature of the system.

If the analyzer and polariser positions are kept fixed and the incident angle is increased the shape of the responsivity curve remains essentially unchanged, however, the peak position displaces to higher values of refractive index as shown in Fig. S-1 in the Supplementary material. The change in peak position for the case shown is approximately 1.39×10^−2^ RIU per degree of incident angle.

We required a dynamic range of approximately 2×10^−2^ RIU, so we were prepared to sacrifice responsivity to achieve a theoretical value of around 170/RIU (blue curve in Fig. 2), this sacrifice increases the dynamic range which allows us to keep the system parameters fixed once aligned and which we demonstrate still gives refractive index sensitivity better than 10^−6^ RIU over the required range. This theoretical responsivity is compared with the experimental value in Section 3.1.

### 2.2 Instrument configuration

We now examine the individual components in Fig. 1. The key element was the custom CMOS modulated light camera which enables the modulated signals at many points to be measured simultaneously; essentially operating as 256 lock in amplifiers in parallel. The requirements of this camera determine the choice of polarization modulator and light source. The custom CMOS camera is a linear array of modified active pixel sensors. Each pixel has four storage capacitors that allow four samples to be taken during each modulation cycle; each capacitor stores electrons over a quarter of a cycle. Each storage capacitor can store approximately 6×10^8^ photoelectrons. This means that if we can approach shot noise limited performance the noise on each pixel is better than 1 part in 10^4^; spatially averaging over several pixels or temporally averaging over many frames allows the signal to be measured with lower noise levels as discussed in Section 2.3. These cameras have been used previously to measure low modulation depth signals (~1 part in 10^6^) in picosecond laser ultrasound (Smith et al., 2010) and spatial modulation microscopy (Fairbairn et al., 2012). In the present case the modulation depths are high (10–50%) but there is a requirement to measure these signals with good signal to noise ratio (SNR); so the demands on the camera are essentially the same. In order for the camera to operate with SNR limited by shot noise it is necessary to fill the storage capacitors to at least 20% of full capacity, this means that in one quarter of a cycle the number of stored photoelectrons needs to match the capacity of the capacitors. Clearly, as the modulation frequency increases for a given power level the number of photoelectrons changes, so to ensure that the capacitors are filled it is necessary to vary the modulation frequency. This, in turn, determines the choice of modulator and light source.

In previous work (Hooper et al., 2006) photoelastic modulators were used to modulate the polarization, these are excellent devices, having a very large clear aperture and are compatible with any light source. However, apart from being rather bulky, they are resonant devices operating at a fixed frequency (typically ~47 kHz). The requirement to fill the storage capacitors means frequency agile device must be used, we therefore used an Electro-Optic Modulator (EOM, Thorlabs EO-AM-NR-C1). A drawback to the EOM is the small clear aperture and long propagation length inside the crystal. This means that using LEDs becomes impractical as most of the light will not pass through the EOM, however, this is not an issue if a laser or Superluminescent Light Emitting Diode (SLED) is used. For this reason the light source chosen was a HeNe laser (MELLES GRIOT 15 mW 633 nm) operating at 633 nm wavelength with maximum output power of 10 mW. The chosen wavelength also gives the desired compromise between sensitivity and dynamic range.

The driving voltage for the EOM was chosen to maximize the phase modulation of the 1*f* signal. After passing through a quarter waveplate which was used to correct any fixed phase offset between the p and s components, the modulated beam emerges linearly polarized with a sinusoidally changing polarization angle as assumed when deriving Eqs. (1)–(3) in Section 2.1. The driving voltage to the EOM was used as the reference to the custom CMOS camera.

The DC, 1*f* amplitude and 1*f* phase can be reconstructed from the four *samples*, *S*_*n*_, using a standard phase stepping algorithm (Fairbairn et al., 2012) shown in Eqs. (4)–(6).

(4) $$\mathrm{DC}=\frac{S_{1}+S_{2}+S_{3}+S_{4}}{4}$$

(5) $$1f\;\mathrm{Amplitude}=\sqrt{{\left(S_{3}-S_{1}\right)}^{2}+{\left(S_{4}-S_{2}\right)}^{2}}$$

(6) $$1f\;\mathrm{Phase}=\arctan\left(\frac{S_{3}-S_{1}}{S_{2}-S_{4}}\right)$$

The polarization is modulated in a sinusoidal fashion, however, from the final system Eqs. (1)–(3) above we can see that the response is not perfectly linear especially for large excursions in the modulation, thus leading to a component at harmonics of the fundamental dither frequency i.e the 2*f* term (Eq. (3)). The four phase steps used in the camera architecture mean that these second harmonic signals are not detected, as terms that are in quadrature for the fundamental are in antiphase for the harmonic and thus cancel.

Unlike in previous versions of the camera (Fairbairn et al., 2012), the camera is now a fully plug and play device; the camera unit contains the photo sensitive chip, the FPGA used for driving the device, and an onboard 16 bit ADC. The digitized data is sent over USB to the PC. More details concerning the camera technology will be the subject of a future publication.

After the quarter wave plate the beam was expanded by lens 1&2 and directed to the fixed 1:1 imaging arm (lens 3&4) by a dielectric mirror (Edmund NT45-995) on a precision rotation stage (Newport UTR80-S). This rotation stage sets the incidence angle of the beam on the gold surface (gold thickness was 50±2 nm, deposited on a BK7 cover glass (Fisher FB58620) using sputterer from (EMITECH K575X) and was chosen to be at the optimal part of the plasmon dip. The 1:1 imaging arm reimages the center of rotation of the mirror to the gold surface which ensures that the beam does not move on the sample surface when the incident angle changes.

The prism (Edmund NT65-591) is made from SF11 glass, (refractive index 1.78 at 633 nm); this reduces the required mounting angles as the plasmon angle was ~54° compared to ~70° with a BK7 prism. This aids compactness and alignment.

After reflecting from the gold coated cover slip and passing through the analyzer, the light is reimaged, and slightly demagnified on the custom CMOS detector by lenses 5 and 6.

The sample was illuminated over a 10 mm×10 mm area, and the linear camera records a single line through this area. The direction of this line depends on the orientation of the flow cell (a custom flow cell made of polyoxymethylene (delrin) with two channels with a center to center separation of 3 mm, and the size of each channel is 1.6 mm×8.6 mm×0.5 mm) channels used in the experiment, shown in the inset to Fig. 1. The two channels can be used independently or connected in series. Multiple analytes can be assessed along the line D.

### 2.3 Preparation of antibody coated samples

Antibody attachment was carried out with commercially available self-assembled monolayer-coated gold coverslips (10% COOH-(PEG)6-C11-SH, 90% OH-(PEG)3-C11-SH; Reichert Inc., USA) activated with 1-Ethyl-3-(3-dimethylaminopropyl) carbodiimide (0.2 M) and N-hydroxy succinimide (0.1 M).

An anti-fibrinogen antibody (Abcam; ab10066) diluted to 20 µg/ml in sodium acetate buffer pH 5.2 was coupled to one channel (channel A, inset Fig. 1) whilst the other channel (channel B) was used as a reference. Following antibody coupling, any remaining active surface was deactivated with ethanolamine to prevent attachment of protein during the subsequent binding reactions. The process of antibody attachment typically involved a change of 1000 µRIU and the index change can be used as a measure of the quality of the attachment process.

The multiple spot slides were prepared in the same way as above, using the same commercial slides and chemicals, except that the whole slide was activated and only two drops of antibody solution (anti-fibrinogen antibody (Abcam; ab10066) diluted to 100 µg/ml in sodium acetate buffer pH 5.2 containing 20% glycerol) were deposited on the surface. The drops were left for 2 h and then washed off the slide with blocking solution (ethanolamine) and rinsed in Phosphate Buffered Saline-Tween20 (PBST).

### 2.4 Preparation of protein samples.

Fibrinogen solutions used in the experiments were prepared to a concentration of 5 µg/ml in PBST.

## 3 Results and discussion

### 3.1 Measurement of system responsivity and sensitivity

Firstly, we discuss the change in signal level for a change in sample RIU (the responsivity). Then we consider the minimum change in RIU that can be detected taking into account the noise in the instrument (the sensitivity). Finally, we reiterate the advantages of using a reference channel to reduce the influence of long term drift.

**Fig. 3:**
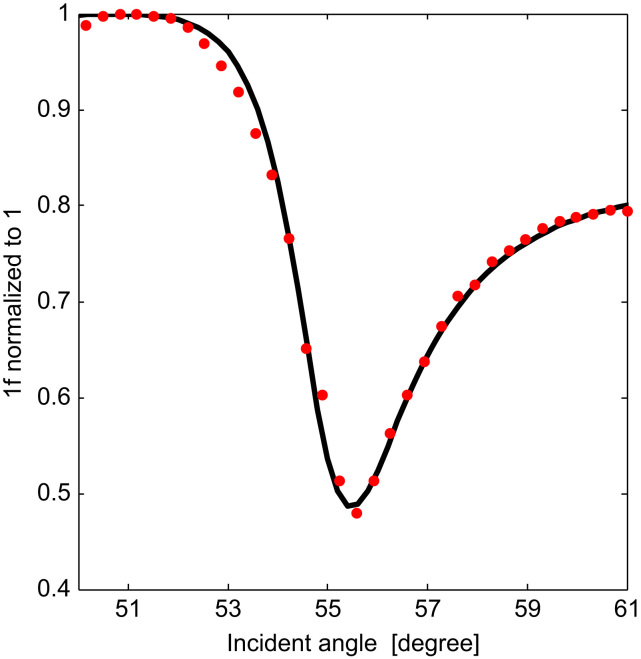
*1f* vs. incident angle at sample refractive index of 1.34, for fixed polarizer and analyzer angles (−6° and 1° respectively). Red dots represent experimental data, while the black curve shows the simulated values. (For interpretation of the references to color in this figure legend, the reader is referred to the web version of this article.)

The input polarization, which in this case was required to be vertical (p), was determined by the EOM crystal angle. The angle of the analyzer was selected to give the maximum light intensity. The incident angle at the glass/gold interface, which was controlled by the rotation of a dielectric mirror, was optimized by detecting the 1*f* amplitude across an incident angle scan range of 10°. Fig. 3 shows the recovered 1*f* signal on a sample index of 1.34. The incident angle was set at the angle where the gradient of the curve is at a maximum, in this case at 54.5°.

The modulation frequency can be adjusted according to the intensity of light incident on the camera. In general, operating at higher modulation frequencies reduces the noise, such as laser fluctuations, although sufficient light must fall on the camera to fill the well to ensure shot noise operation. The incident angle was adjusted so that the maximum sensitivity occurred at a refractive index of 1.34.

The flow cell was aligned as shown in the inset to Fig. 1. The reference channel A was injected with water, while signal channel B was filled with samples of different refractive indices. The solutions were made from Isopropyl Alcohol (IPA) and distilled water in different proportions, whose refractive is found in Glover and Goulden (1963).

We examined the variation of modulation signal for varying refractive index to demonstrate the obtained responsivity and the uniformity of the response across pixels as shown in Fig. S-2 and S-3 in Supplementary information.

We determined the responsivity to be 150/RIU which is quite close to the simulation results shown in Fig. 2. The differences are primarily due to uncertainty in the dielectric properties and thickness of the gold film.

### 3.2 Noise analysis

The minimum detectable change in RIU depends on both the responsivity and the noise in the instrument.

**Fig. 4:**
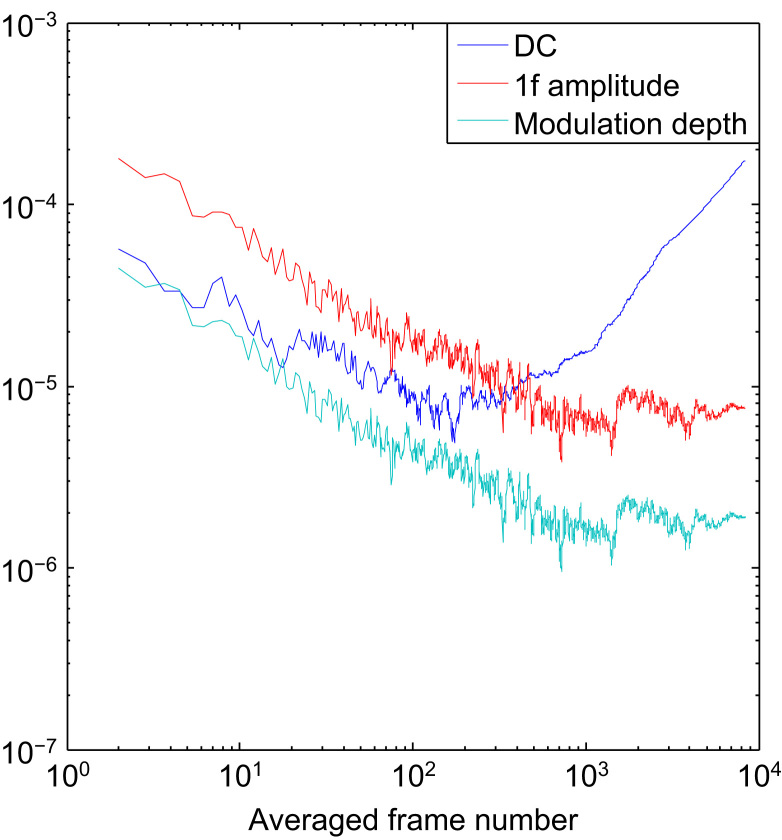
Dark noise of the custom CMOS camera. Here the modulation depth is calculated with the equivalent DC at which the camera was illuminated to saturation.

We firstly assess the dark noise from the camera. Fig. 4 shows dark noise for the DC, amplitude of the 1*f* signal and the modulation depth. At around 200 averages the DC noise increases due to drift and at 2000 averages the modulation depth noise starts to flatten out, this is the noise floor of the data acquisition system and is the level at which the performance of the system cannot be improved by averaging – this is the ultimate performance limit of the current system. The limitation appears to be caused by a small residual amount of coherent noise in the chip. This corresponds to a minimum modulation depth of 9.6×10^−7^ and taking into account the responsivity of 150/RIU shown earlier this to leads to an ultimate limit of detection of 6×10^−9^ RIU at a single pixel without spatial averaging.

In practice, if we wish to make measurements quickly it is not possible to average indefinitely so the number of averages was limited to the number obtainable in 1 s. This gives sufficient bandwidth to measure binding kinetics. The camera currently transfers around 640 frames per second to the computer. This is a limitation caused by the method of transferring the data over USB which could be improved at a later date. To obtain one measurement per second we are therefore limited to 640 averages. This reduces the sensitivity to 1.8×10^−6^ in modulation depth and therefore 1.2×10^−8^ RIU.

The camera and data acquisition system is not the only source of noise, there is also noise from the laser and the environment. This limits the actual system performance. The noise level of the modulation depth, after taking 640 averages, is 4.4×10^−5^ which corresponds to a system sensitivity of 3×10^−7^ RIU. This sets the practical sensitivity limit of the current system. While this is a good level of sensitivity it can be further improved as discussed in the conclusion, it is clear that, apart from the acquisition rate, the camera system does not impose a limit of the achievable performance. Sensitivity better than 10^−6^ RIU was achieved over the whole refractive index range of 2×10^−2^ RIU, this sensitivity takes into account any changes in responsivity and measured noise levels for different index points.

### 3.3 Antibody–protein binding experiments

**Fig. 5:**
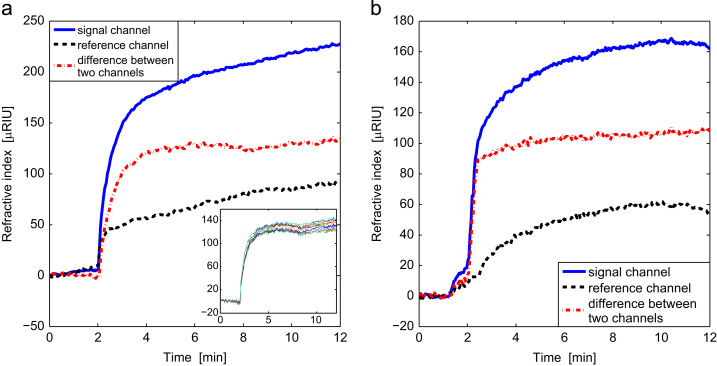
(a) Traces of the signal channel (blue lines) and reference channel (black lines), and the difference between the two channels (red lines) detected by (a) our system and (b) Reichert SR7000DC SPR system. Calibration with the reference channel removes the refractive index increase caused by non-specific binding, and both systems detected similar level of index change induced by specific binding. The subfigure in (a) shows the traces of ten pixels in the signal channel, which demonstrates the consistent performance of this system in multi-point detection. (For interpretation of the references to color in this figure legend, the reader is referred to the web version of this article.)

The instrument has measured real time protein to antibody binding, the sample slide was setup as described in Section 2. Channel A was used for signal and a specific antibody was attached to the gold surface, while channel B was used for reference. A fibrinogen protein solution (concentration of 5 μg/ml) was passed through both channels. In order to obtain the results in Fig. 5(a) we averaged the results over 20 pixels in the flow channels A and B and plot the difference between the signal and reference channels. (The results for the individual pixels are show in Fig. S-4 in the Supplementary information.) These traces are compared to the results of a binding test repeated with the same concentration of protein on a commercial single point measurement SPR system (Reichert SR7000DC).

After two minutes the protein starts to bind to the surface. After a further two minutes saturation of the antibody binding sites is approached. The reference and measurement channel signals both continue to increase due to non-specific binding which is similar in each channel.

To show the consistency of this system, the binding rates of 20 pixels in the signal channel were calculated by fitting to the simple exponential binding model, which ignores dissociation,

(7) $$Signal=A(1-e^{-bt})$$

where A is the signal at *t*=*∞*, *b* is the binding rate constant and *t* is time. By exponential fitting to the binding curves, the mean value of b was 0.030 s^−1^ with a standard deviation between pixels of 0.0030 s^−1^. This confirms that each pixel is capable of consistent independent measurements. The uncertainty in the determination of the rate constant may be reduced by averaging over each binding site in the present case the effective standard error can be reduced by approximately a factor of $\sqrt{20}$.

The change in RIU obtained with the Reichert instrument was slightly lower than the results obtained here, presumably due to the different levels of antibody attachment achieved during the surface preparation step.

In order to evaluate the potential of the system to visualize multiple binding sites, two spots of anti-Fibrinogen were prepared as described earlier and aligned along a single channel (channel A) in Fig. 1 inset while the linear camera (line D) was aligned parallel to the channel instead of across two channels. The two spots are shown schematically in channel A. The binding curves at each active pixel are presented in Fig. 5a, in which the blue lines and black lines represent the signal change within the two antibody spots and the green lines represent the reference area between these two antibody spots. The variation in the absolute values of the binding curves within the two antibody spots were consistent with the antibody attachment process where we expect less binding at the edges of the channel due to the fact that the samples were deposited by placing droplets over the channels, so we expect to see less antibody attachment at the edges. This assertion is borne out from the measurements of refractive index change obtained during the attachment process which clearly showed greater concentrations of antibody at the center of the channel. Again we used Eq. (7) to exponentially fit to the binding curves. The mean values of the binding rate constant, *b*, of the two antibody droplets were 0.0050 s^−1^ and 0.0056 s^−1^, respectively with standard deviations of *b* were 6.6×10^−4^ s^−1^ and 8.2×10^−4^ s^−1^, respectively. The variation that leads to the standard deviations above does not appear to be due to a simple (i.e. random) statistical variation as we discuss below.

**Fig. 6:**
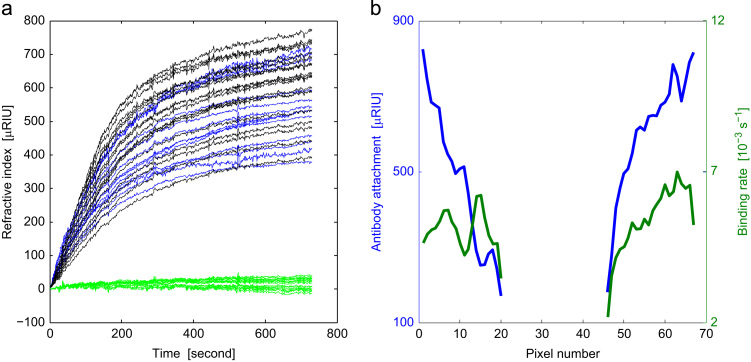
(a): Binding curves from the two antibody spots (blue and black lines) and reference area (green lines). The variation in the final binding level was caused by differences in the antibody attachment across each droplet. The signal variation in the reference area was due to drift and non-specific binding; (b) the blue curve is the antibody attachment level from the fit to the exponential curve, *A*_*exp*_, using Eq. (7) and the green curve is the calculated binding rate from the same exponential fitting. (For interpretation of the references to color in this figure legend, the reader is referred to the web version of this article.)

The pixelated nature of the detection allows us to examine spatial variations in the binding. There was clear evidence in Fig. 6b that the binding rate constant, *b*, correlates to the level of antibody attachment. The quality of the fit to the exponential curve does not change systematically with the position of the detected spot. The multipoint detection thus reveals systematic changes in the binding rate that are correlated to the amount of antibody attachment. The precise reason for this effect requires further investigation but it is possible that when the level of antibody attachment is low the alignment of the antibodies are less ordered so that they provide a less favorable orientation to the binding proteins. Whereas with a more heavily populated spot the proximity of the bound antibodies means they tend to project normal to the array surface. Whatever, the mechanism for this effect a single point detector would simply average this variation which would not be observed.

## 4 Conclusions

The phase sensitive SPR system using a custom CMOS camera gave an optimum refractive index sensitivity of 3×10^−7^ RIU within a refractive index range of 2×10^−2^ RIU. Sensitivity better than 10^−6^ RIU was achieved over the whole refractive index range of 2×10^−2^ RIU.

The advantages of this technique can be summarized as follows:

• The detector methodology allows one to operate at a single angle of incidence for the whole measurement. This is especially suited for imaging where varying the incident angle or incident wavelength is problematic.
• The detector array facilitates this measurement because it is not readily practical to use a conventional detector to detect modulated light at a reasonable modulation frequency.
• The detection methodology allows one to use the polarization dithering technique that is extremely difficult to implement with a conventional array detector such as a CCD.
• Operation at high modulation frequencies is possible using this approach which reduces the effect of microphonic noise allowing one to operate in more hostile environments.
• The method allows one to trade sensitivity for dynamic range by altering the polarizing components only, making for a highly versatile instrument.

The linear camera system used here is ideal for examining binding across flow channels and the deep well depth gives great sensitivity. The sensitivity in the present system is limited by the optical system rather than the camera performance. To measure binding on multiple spots a 2D array is more suitable and a lock in camera array matched to this format is presently being tested; in this case the well depth is rather smaller and we need to evaluate whether the sensitivity is still limited by the optical system.

Finally, it is possible to obtain greater sensitivity with this system, albeit at the expense of reduced dynamic range. From our simulations, which agree well with experiments, we expect a responsivity of 940/RIU can be obtained, which is more than 5 times greater than the current responsivity of 170/RIU. For similar noise levels this means a refractive index sensitivity of 4.7×10^−8^ RIU, with very low dynamic range of 2×10^−3^ RIU. In the longer term one could devise an adaptive system where the polarizer and analyzer were adjusted for large dynamic range, to find the background refractive index, after which the system could then be optimized for sensitivity around this background.
