# Supplementary material for: Highly sensitive multipoint real-time kinetic detection of Surface Plasmon bioanalytes with custom CMOS cameras
Source: Biosens Bioelectron. 2014 Aug 15;58(100):157–64. doi: 10.1016/j.bios.2014.02.042 (PMC4009403; doi:10.1016/j.bios.2014.02.042)
Supplement: Supplementary file 1 — Supplementary data [file mmc1.docx]

Supplementary information

Figure S-1 shows a series of curves for the same setting of the analyzer and polarizer with a variation of the incident angle. In this case we see that the responsivity and dynamic range hardly change while refractive index values corresponding to maximum sensitivity increases with incident angle.


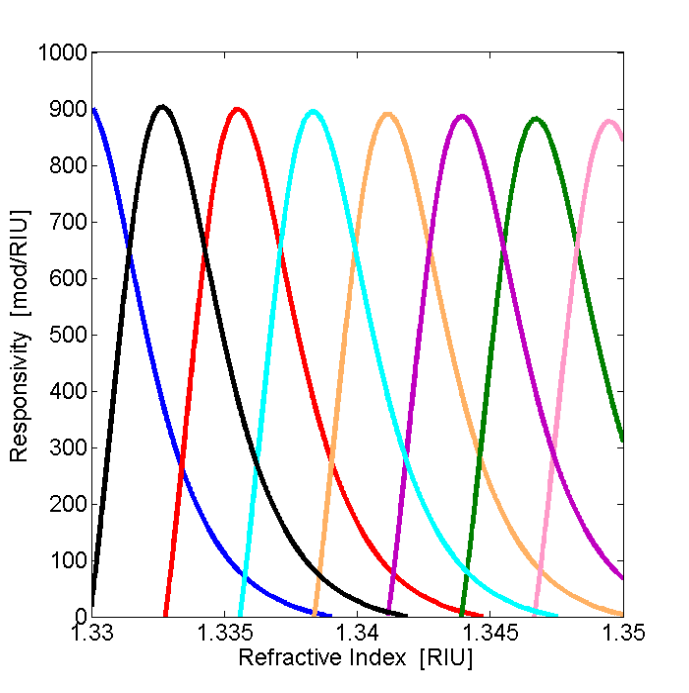


Figure S-1. Responsivity using different incident angles from 54.5 deg. to 55.9 deg. from left to right, in increments of 0.2 deg. The polarizer and analyzer angles were 30 and 166 degrees respectively for all incident angles. The position of maximum modulation depth in refractive index shifts from 1.33 to 1.35 with little change to the maximum value and dynamic range.

Figure S-2 shows the response from the camera with changing sample index. There are 39 pixels in both channels, which correspond to an illumination length of approximately 1.5mm. The modulation depth increases as the sample refractive index increases from 1.33 to 1.35. The reference channel remained approximately flat. The response from the pixels in the signal channel is very uniform and the response from a single pixel in the signal channel is shown in c showing the change in signal level for each change in sample index.


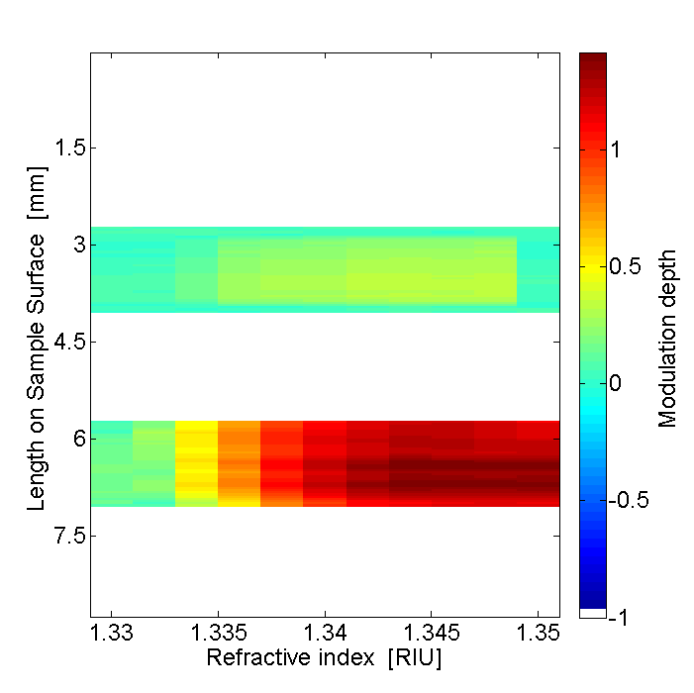


Figure S-2 Modulation depth vs. refractive index across the linear camera at a fixed incident, polarizer and analyzer angles. The vertical direction indicates the position on the sample, the 256 pixels of the camera correspond to a length of 8.7mm. The reference and signal channel are located at the 3 and 6mm positions, respectively. Samples with different refractive indices were passed through the signal channel while distilled water flowed through the reference channel causing the large change in signal in the signal channel

From Figure S-3 we can determine the responsivity to be 150/RIU. This is quite close to the simulation results of 17-0 /RIU. The differences are primarily due to uncertainty in the dielectric properties and thickness of the gold film.


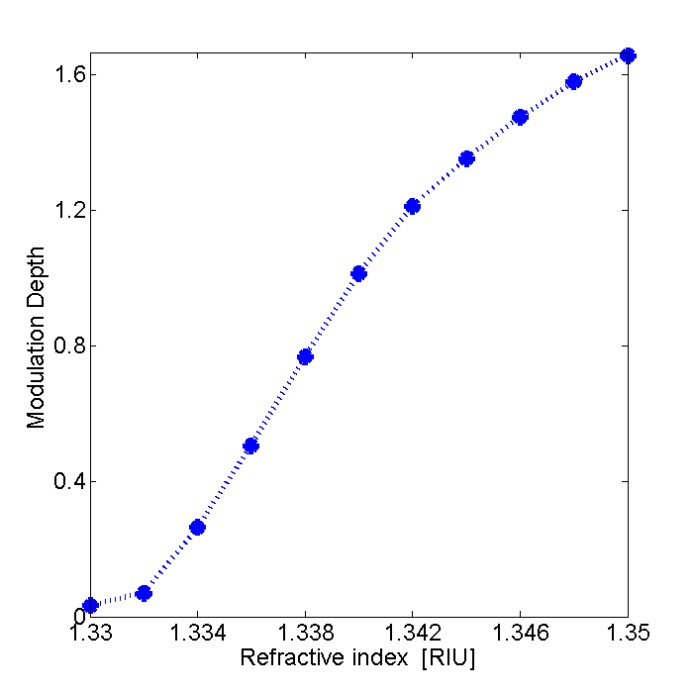


Figure S-3 The modulation depth of a single pixel in the signal channel.

Figure S-4 shows 20 pixels for both the signal (a) and reference (b) channels. In this configuration there were approximately 40 pixels in both channels, however, the central 20 pixels in each channel were used to analyze binding in order to eliminate non-ideal liquid flow at the edges of channels. The response across the signal channel is very uniform and shows an exponential increase as the protein binds to the antibody. The reference channel has more variation and shows different rates of nonspecific binding of the protein to the SAM.


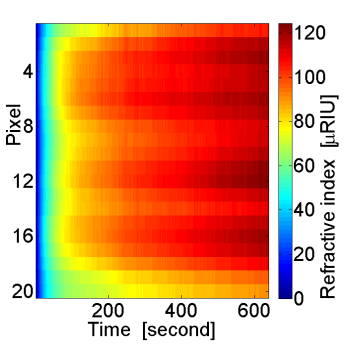

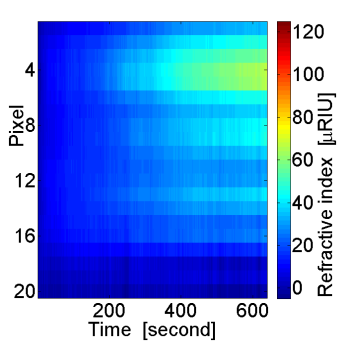


(a) (b)

Figure S-4 Traces in μRIU of Fibrinogen antibody-protein binding step in the signal channel (a) and reference channel (b). The antibodies were immobilized in the signal channel only, the reference channel contained the de-activated SAM surface. 5 μg/ml protein was flowed through both channels at a rate of 20 µl/min. The significant increase of the refractive index in the signal channel (a) corresponds to the antibody-protein binding and any non-specific binding, while the slight increase of refractive index in the reference channel (b) corresponds to the non-specific binding only.
