# Supplementary figures and images for: Highly sensitive multipoint real-time kinetic detection of Surface Plasmon bioanalytes with custom CMOS cameras
Source: Biosens Bioelectron. 2014 Aug 15;58(100):157–64. doi: 10.1016/j.bios.2014.02.042 (PMC4009403; doi:10.1016/j.bios.2014.02.042)

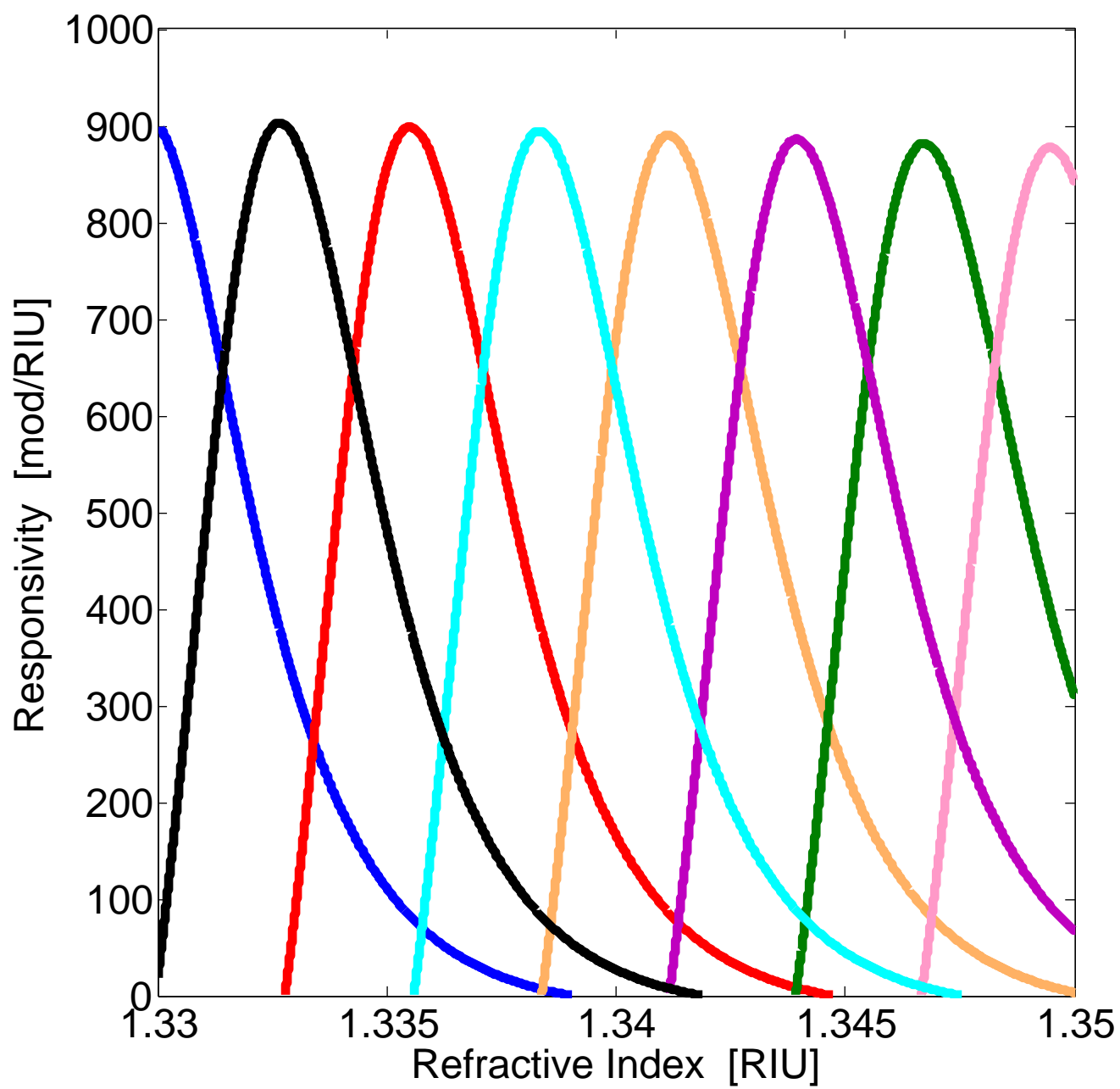

Supplement: Supplementary file 2 — Supplementary data [file mmc2.pdf]

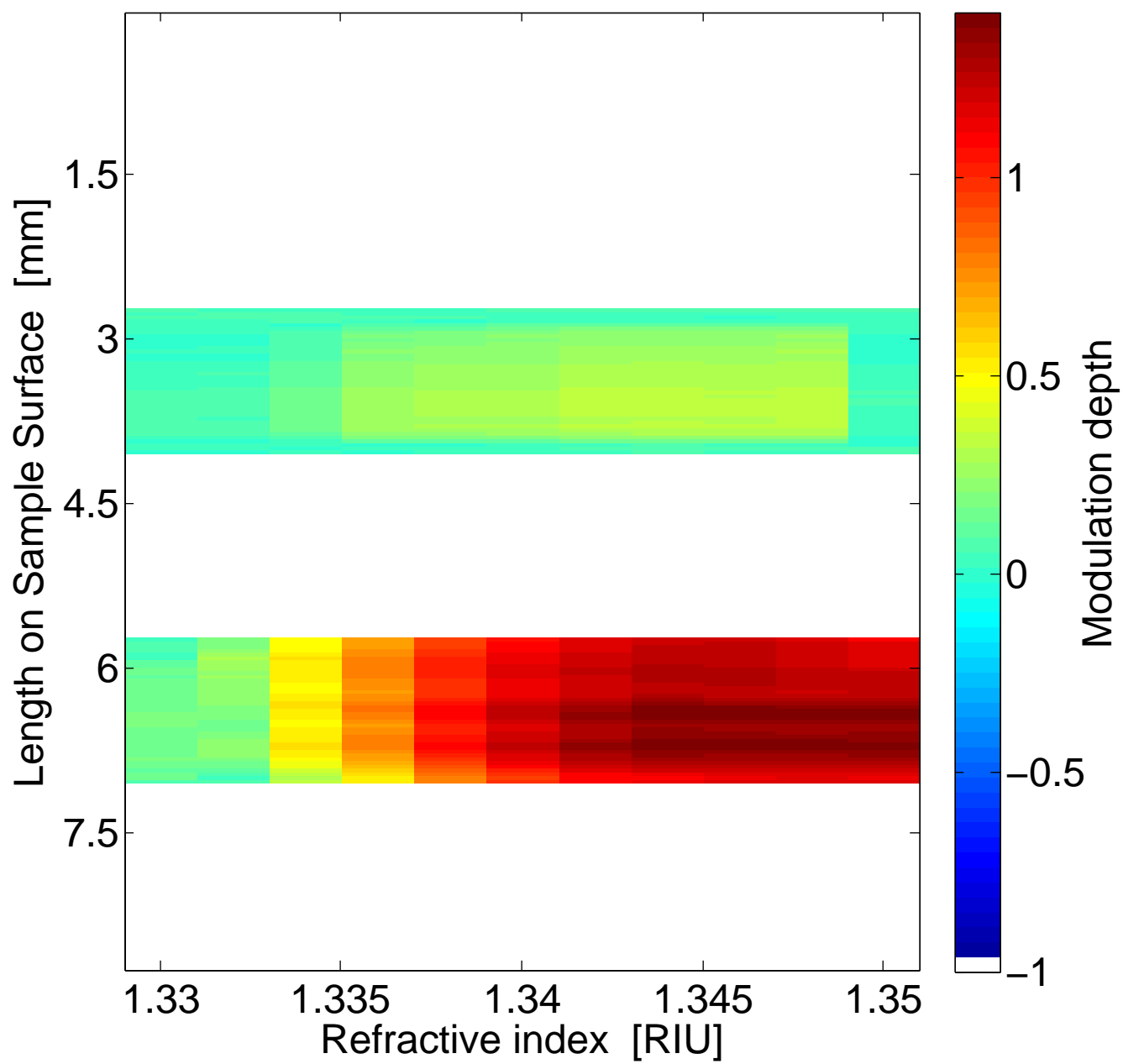

Supplement: Supplementary file 3 — Supplementary data [file mmc3.pdf]

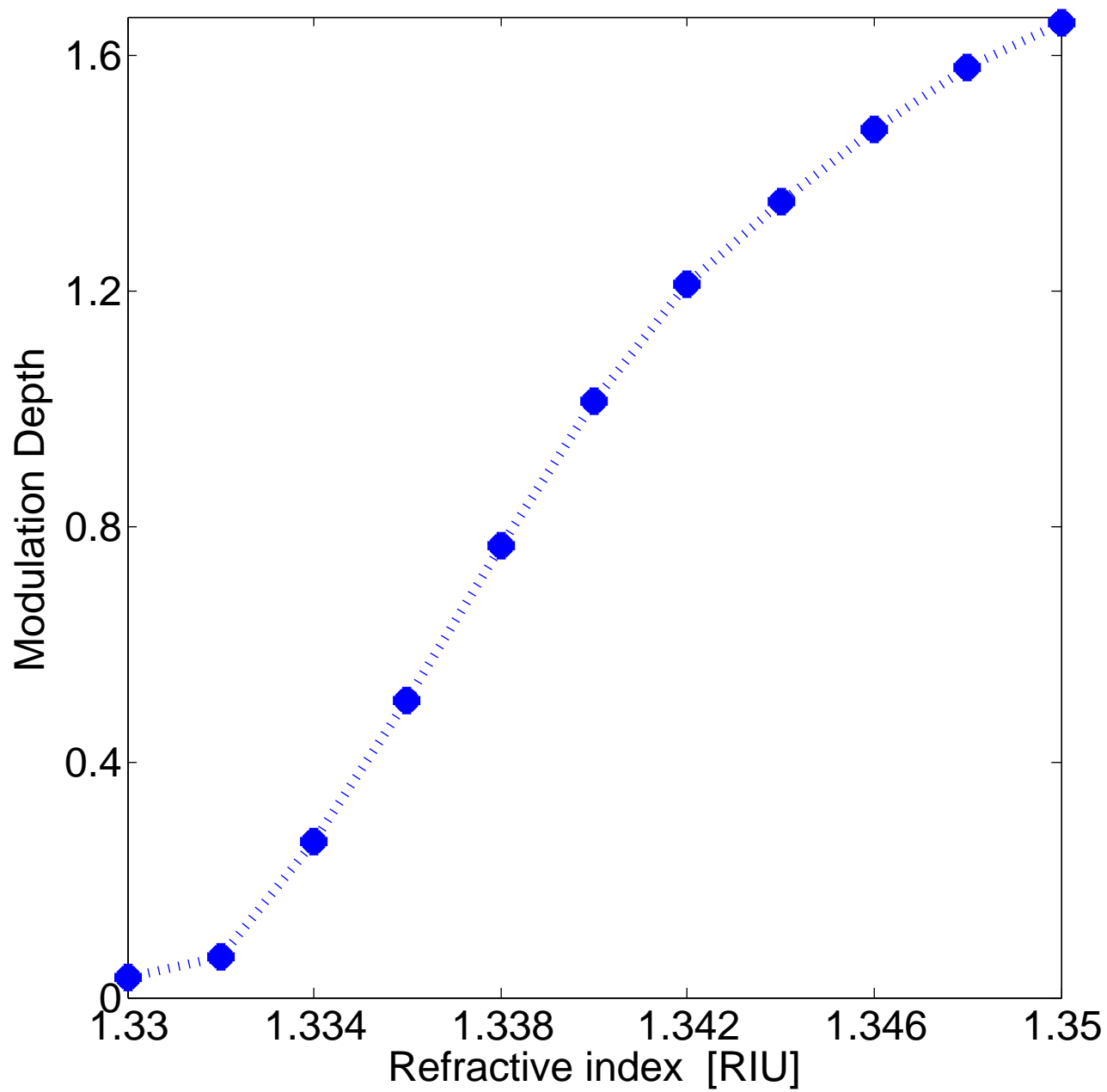

Supplement: Supplementary file 4 — Supplementary data [file mmc4.pdf]

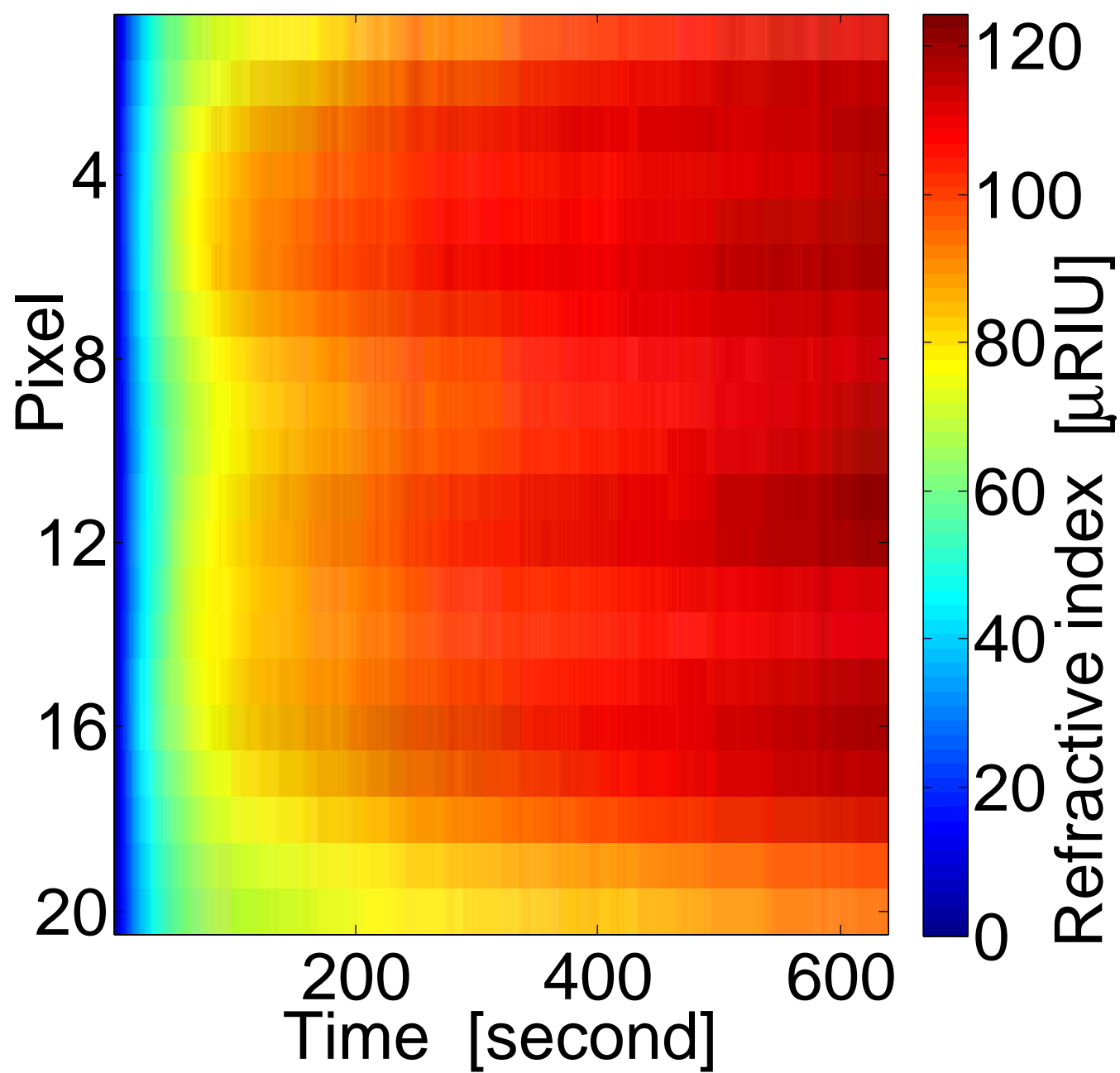

Supplement: Supplementary file 5 — Supplementary data [file mmc5.pdf]

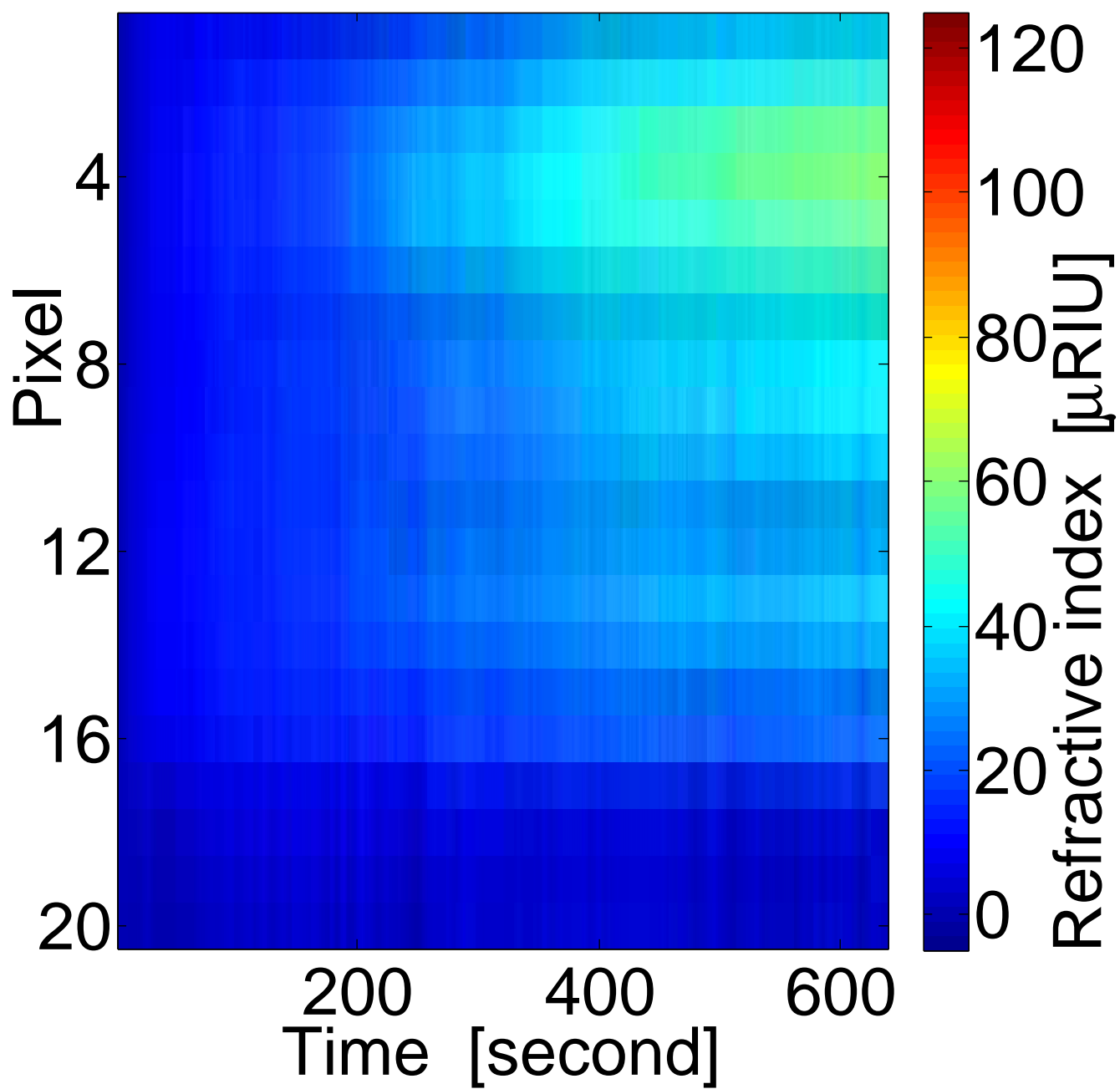

Supplement: Supplementary file 6 — Supplementary data [file mmc6.pdf]
